# Aclarubicin disrupts RNA polymerase II progression at replication-coupled histone genes

**DOI:** 10.64898/2026.07.08.737292

**Published:** 2026-10-24

**Authors:** Kevin K. Nguyen, Matthew Wooten, Kami Ahmad, Steven Henikoff

## Abstract

Anthracyclines are effective chemotherapeutic agents that cause DNA and chromatin damage. One member of the anthracyclines, aclarubicin, has gained therapeutic interest due to its ability to kill cancer cells through chromatin-based mechanisms, thus avoiding the off-target effects associated with DNA damage. However, the molecular mechanism of action leading to aclarubicin-induced chromatin damage remains elusive. Here we performed Cleavage Under Targets and Tagmentation (CUT&Tag) of RNA polymerase II (Pol II) and other transcriptional regulators in human cells following aclarubicin treatment. We found that aclarubicin strongly disrupts the replication-coupled histone genes, resulting in a nonproductive accumulation of Pol II transcription machinery here beyond the levels at other genes. We attribute this sensitivity to the dense Pol II loading and rapid transcription of the histone genes, which intensify the chromatin-disrupting effects of aclarubicin. Together, our findings shed light on the mechanism of aclarubicin as an anticancer drug and point to the replication-coupled histone genes as a target for therapeutic intervention.

